# Artificial intelligence in medico-dental diagnostics of the face: a narrative review of opportunities and challenges

**DOI:** 10.1007/s00784-022-04724-2

**Published:** 2022-09-24

**Authors:** Raphael Patcas, Michael M. Bornstein, Marc A. Schätzle, Radu Timofte

**Affiliations:** 1 Clinic of Orthodontics and Pediatric Dentistry, Center of Dental Medicine, University of Zurich, Zurich, Switzerland; 2 Department of Oral Health & Medicine, University Center for Dental Medicine Basel UZB, University of Basel, Basel, Switzerland; 3 Computer Vision Laboratory, Department of Information Technology and Electrical Engineering, ETH Zurich, Zurich, Switzerland; 4 Computer Vision Laboratory, CAIDAS and Institute of Computer Science, Faculty of Mathematics and Computer Science, University of Wurzburg, Wurzburg, Germany

**Keywords:** Artificial intelligence, Photography, Face, Neural networks, Government regulation and oversight

## Abstract

**Objectives:**

This review aims to share the current developments of artificial intelligence (AI) solutions in the field of medico-dental diagnostics of the face. The primary focus of this review is to present the applicability of artificial neural networks (ANN) to interpret medical images, together with the associated opportunities, obstacles, and ethico-legal concerns.

**Material and methods:**

Narrative literature review.

**Results:**

Narrative literature review.

**Conclusion:**

Curated facial images are widely available and easily accessible and are as such particularly suitable big data for ANN training. New AI solutions have the potential to change contemporary dentistry by optimizing existing processes and enriching dental care with the introduction of new tools for assessment or treatment planning. The analyses of health-related big data may also contribute to revolutionize personalized medicine through the detection of previously unknown associations. In regard to facial images, advances in medico-dental AI-based diagnostics include software solutions for the detection and classification of pathologies, for rating attractiveness and for the prediction of age or gender. In order for an ANN to be suitable for medical diagnostics of the face, the arising challenges regarding computation and management of the software are discussed, with special emphasis on the use of non-medical big data for ANN training. The legal and ethical ramifications of feeding patients’ facial images to a neural network for diagnostic purposes are related to patient consent, data privacy, data security, liability, and intellectual property. Current ethico-legal regulation practices seem incapable of addressing all concerns and ensuring accountability.

**Clinical significance:**

While this review confirms the many benefits derived from AI solutions used for the diagnosis of medical images, it highlights the evident lack of regulatory oversight, the urgent need to establish licensing protocols, and the imperative to investigate the moral quality of new norms set with the implementation of AI applications in medico-dental diagnostics.

## Introduction

### The face as an object of diagnostic interest

Interest in the analysis of the face is apparent in the earliest records of medical art. The E. Smith Papyrus, dating c. 1600 before the Common Era and as such the most ancient known medical text, opens with a diagnostic description of 15 injuries of the face. Since the preserved Papyrus only illustrates a total of 48 case histories, emphasis on the examination of the face, which in ancient Egypt included visual, tactile, and also olfactory clues, is evident [1, 2].

In the context of dental medicine in general, and orthodontic or maxillofacial surgery in particular, the analysis of the face has enjoyed great interest, both for diagnostic purposes and for outcome assessments. The information contained within a facial image is manifold and includes evidence on possible pathologies, malformations, or deviations of the norm, be it of the skin or of underlying musculoskeletal structures, that may all be instrumental in the diagnostic analysis of a case. The face mirrors, on the other hand, the aesthetic success of an orthodontic or maxillofacial intervention, and judgment is made on whether a balanced and harmonious facial appearance is restored or established.

Yet, the analysis of the face has remained predominantly a *clinical* task that depends largely on the clinician’s expertise and experience, and the assessment of the aesthetic outcome suffers of inconsistency owing to *subjectivity*. Medical sciences have thus since long attempted to infer information contained in medical images of the human face in a structured and dependable approach, in order to overcome the shortcomings related to subjectivity or the dependency of expertise and experience.

Applying artificial intelligence (AI) to medical diagnostics and outcome evaluation would seem to satisfy these demands. With the intention to reproduce the human cognitive process, AI has the potential to entirely modify dental clinical care [3] and research [4]. Presenting the potentials of AI application in medico-dental diagnostics of the face, this review ventures to share—*from the vantage point of a practitioner*—current developments of AI in this field, present possible novel opportunities, and discuss prevalent obstacles.

### AI and mimicking natural intelligence

**Fig. 1:**
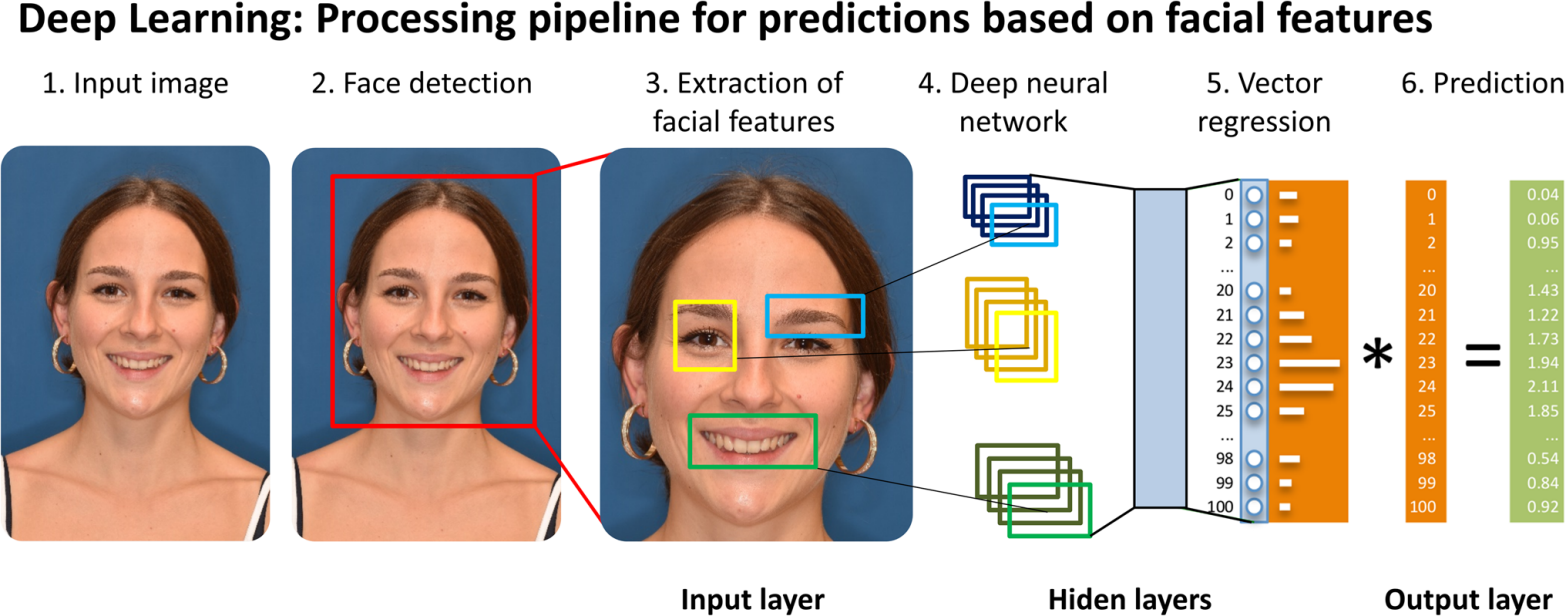
Typically, for (1) an input image, (2) the face is first detected and fed to (3–6) a deep learning model that (3) extracts facial features and (4) assesses the relationships between them and annotated data to (5) regress to a range of values that it is finally (6) mapped to a predicted label output

Natural intelligence is based on a learning process where a stimulus is first perceived, then its attributes interpreted. The interpretation of the stimulus leads, in an intelligent *living being*, to a response in terms of an adaptation when exposed to the same stimulus again. AI can be understood in a similar vein: data serve as stimulus, and patterns within the data are being recognized, extracted, and interpreted. Deep learning is the part of machine learning that aims to emulate the natural learning process by use of artificial neural networks (ANN) which are based on multiple layers trained to progressively extract higher-level features (Fig. 1). These convolutional neural networks are patterned loosely on the mammalian visual cortex [5] and will, this time for an intelligent *agent*, likewise lead as part of a learning process to an adaptation when exposed to the same stimulus again. In medical imaging, the triplet terminology of stimulus/pattern recognition/interpretation would thus be reiterated as data/findings/diagnostics.

In dental medicine, the stimuli are mostly data originating from images or patient files, yet some research have also used machine learning to find associations to olfactory patterns such as oral malodour [6].

### Data acquisition and curation

Photographic images, which are being used as data for facial diagnostics, either are produced a priori as dedicated medical facial images or consist of re-utilized non-medical images. While customized medical facial images are generated under tailored and standardized conditions, the use of non-medical data allows to access an immense amount of commercially collected data. Obviously, the origin of the facial images will have considerable implications on research design and ethical considerations. Some of them will be discussed below.

Before data can be fed to a neural network, they must be curated. Data curation involves the organization and annotation of the information contained within the data. More specifically, data curation in medicine focuses on extracting relevant information from patient history or medical images. One of the prime factors of driving the applicability of AI and deep learning in medical sciences at such incredible pace is surely the fact that digitization of health-related records has become the norm, which facilitates data curation immensely. Patient history and imaging (radiographic, histologic, and others) are usually not only produced in a digital format, but often also even labeled or categorized as they are being created [5]. This is especially true for the face, for which established standardized high-resolution 2D-photography has been complemented by modern 3D acquisition techniques such as stereophotogrammetry, laser scanning, structured light scanning, or 3D image reconstructions of CT or CBCT data [7, 8]. (A comparative analysis on the diagnostic performance of the different image acquisition types would indeed be valuable, but the seeming lack of scientific literature on this subject precludes any detailed comparison.)

Acquisition and storage of health-related digital data were immensely facilitated in the last two decades, as computational devices became ubiquitous, interlinked, and more potent [5]. These winning conditions led the foundation for a remarkable growth in the volume of information. Such “big data” are, however, too large or too complex to be dealt with by traditional data-processing application software [3], and only the evolution of powerful capabilities in machine learning as enabled AI to process and analyze such vast and multidimensional data [9].

With the introduction of AI in medicine, many possibilities and challenges have emerged. It is not the aim of the review to debate on all possible opportunities and eventualities, but rather focus on the predominant benefits and acute concerns related to the use of deep learning in facial image analysis in a medico-dental setting.

## AI applications in medico-dental diagnostics of the face: opportunities

### Deep learning perfectly suited to diagnose facial images

Not all machine learning approaches are suitable for medical diagnostics, but deep learning models based on artificial neural networks generated an intuitive drawing power to health-related, image-based applications, given their apparent strengths in processing big data, pattern recognition, and predictive model building from large high-dimensional data sets [5], and have shown from their very onset outstanding performance in image processing [10]. Particularly for facial recognition, artificial neural networks (ANN) have proven to perform computation-intensive operations based on massive data sets with significant accuracy and performance [11].

Over the last few years, researchers have advanced the implementation of ANN-based predictions in medico-dental diagnostics of the face. The applications in facial diagnostics include predictions for classification of skin pathologies [12–14] or dysmorphic features [15–19], attractiveness [4, 20–22], perceived age [4, 20, 23], or gender [24]. ANN have also been trained to detect health-related patterns and traits in patients, such as pain [25, 26] or stress [27]. These and many more have the potential to alter the healing arts by providing the following benefits to medico-dental care:

#### Optimized care

Deep learning techniques applied to identify or classify patterns in a facial image as a *diagnostic task* have often performed on par or better than professionals [18]. Using neural networks, for instance to classify pathologies or assess attractiveness, is expected to streamline care and relieve the dental workforce from laborious routine tasks [28]. Optimizing diagnostic processes will surely result in faster and more reliable health care, and may hopefully help reduce costs and make inexpensive diagnostics accessible to less privileged parts of the globe [28]. Lastly, AI-based diagnostics holds the potential to increase the impartiality in decision-making processes relating to funding bodies (i.e., insurances, health services).

#### Enriched care

The historical approaches to scoring facial attractiveness of patients have frequently—and rightfully—been disputed [20]. Professional appraisal of attractiveness performed by dentists or surgeon rely on taught rules of an ideal beauty, which often do not concur with the laymen’s or patient’s impression [20]. Moreover, many studies present panel-based assessments, which remain unavailable for the individual. Neural networks based on big data appear to be a promising tool to render additional and novel assessment methods of facial features, on an individual level, available. Sometimes, the processing pipeline will enable to produce a visualization of an outcome. Being able to score the degree of feminization after surgery, or to quantify the changes in age appearance or attractiveness, on an individual basis for each patient—independently of panels and not subject to the clinician’s expertise and experience—will indeed make health care safer, more personalized, and consolidate the participatory paradigm [5].

#### Revolutionized care

**Fig. 2:**
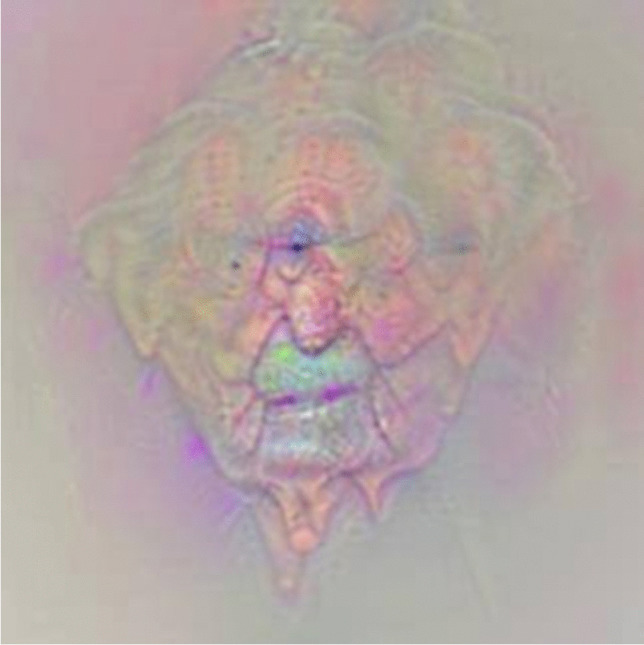
An image generated through deep dreaming: a neural network for facial attractiveness is run in reverse, and activated features related to facial attractiveness are visualized, without actual input image

**Fig. 3:**
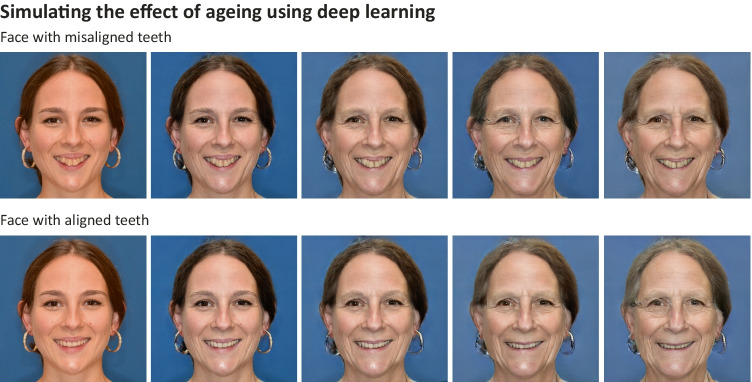
Given any input image of a face, aging predictions can be made using deep learning models (https://arxiv.org/abs/1702.01983). Here, the aging effects of a person are being simulated with aligned and misaligned teeth (the latter using mock-up teeth). Each image shift to the right represents an aging factor of 10 years. Simulating the long-term effect of an intervention may be beneficial to the decision process on whether to undergo a certain treatment or not

Neural networks permit the integration of diverse and complex data. Facial images can be annotated with various information from the patients’ dental or medical history, and then be linked to sociodemographic, social network, or bio-molecular data. New predictions can be conjectured on such vast and varied multi-level data, with the objective to discover new associations [28]. As an example, when ANN based on big data analyzed the outcome of orthognathic surgery, it became apparent that the therapy had a juvenescent effect, and that especially patients undergoing mandibular osteotomies were found to appear younger after the treatment [20]. Neural networks can not only be used to interconnect or link existing variables, but can also be computed to generate a visualization of an inexistent, projected treatment outcome [29, 30]. In what has been coined deep dreaming, trained deep neural networks are run in reverse, being commanded to adjust the original image slightly according to a determined output feature (Fig. 2). A network trained to predict the age can thus be used to modify the input image with exaggerated features related to age that the model learned during training (Fig. 3) [31]. It is therefore expected that AI will eventually facilitate predictive and preventive dental medicine [28], and ultimately be a key factor in the advancement of personalized medicine.

Whatever the degree of change in dental care that may be pursued, the success of AI-assisted diagnostics and treatment planning evidently hinges on its integration in the digital dentistry workflow. It is only with this amalgamation that the desired advancements and improvements in dental medicine can be achieved.

## AI applications in medico-dental diagnostics of the face: challenges

### Transforming commercial AI software of the face to medical AI software

Over the last years, many *commercial* AI solutions for the analysis of the face have emerged, which allow the recognition of gender, age, attractiveness, mood and emotions, or race and ethnicity, all based on a 2D image of the face. Their widespread popularity has led some authors to advocate their use in a medical setting [4, 23, 24, 32]. In contrast, few AI solutions for facial images exist that were designed de novo exclusively for medical diagnostics, which were trained entirely and solely on *medical* images of the face (e.g., for facial palsy [33] or clefts [15] patients). These new solutions are usually less robust, owing to the smaller amount of data available, and often prone to selection bias (e.g., purely based on hospital data) [34]. Most consider therefore the benefit of commercial AI systems based on big data too important to be ignored. AI is the perfect tool to utilize the abundance of available facial images generated from various sources including social networks for predictive analytics. Many times, these facial images are not just available as raw data, but include an annotation for age, gender, or attractiveness, which makes their use even more appropriate. However, the applicability of commercial AI solutions, which are not designed for a medical propose, is on many levels very problematic. The architectural design of commercially available software may not be compatible or ideal for medical diagnostics and therapy assessments [33].

#### Addressing distributional shift

It is evident that the most desirable solution is offered when a network is trained on big data, but then re-trained and refined on medical images of the face. More specifically, when altering AI solutions to obtain a prediction of diagnostic value, the processing pipeline of the network must include training to eliminate or significantly reduce distributional shift, i.e., a mismatch between the data or environment the system is trained on and the data or environment used in operation [35]. AI solutions for attractiveness scoring based on facial images exemplify this issue very well. A commercial AI software trained on social media data originating from a dating platform offers the advantage of using big data, namely millions of individual ratings on a plethora of facial images. This may deliver a reliable portrait of what society currently considers attractive, but it does not necessarily reflect the opinion and judgment of experts. Substantial differences in scoring attractiveness indeed appear to exist between the AI-based prediction and the scores of medical professionals. In fact, even when laypeople (for whom no mismatch would be expected) are given the task to evaluate the attractiveness of faces *in a medical environment*, their score might differ from the AI processed score, too [21].

#### Addressing confounders

Accounting for confounding effects (or biases) is a serious challenge when using deep learning in medical imaging studies [36]. Confounders distort the feature extraction and thus the prediction. Certain distractors can easily be removed by training the network to exclude clues outside the region of interest. Others can be more difficult to filter. For facial images, the issue of emotions illustrates this problem nicely. Facial expressions in general, and emotions in particular, affect facial attractiveness: happiness increases facial attractiveness, and stress will reduce it. While there can be no objection if happiness increases attractiveness on an image featured on social media, in a diagnostic setting, such a distortion of face is considered a transient aspect that must be excluded in order not to impair the accuracy of the evaluation. Re-training a neural network on dedicated medical images taken under a controlled setting supports the network in recognizing emotions as distractors and such a re-training can reduce the bias caused by facial expressions [37].

#### Unsafe failure mode

An AI solution is able to calculate a prediction even in a case where the prediction will not be performed with high accuracy. Insufficient training or missing data can provoke insensible outcomes, and it is therefore of primordial importance that the prediction is supplemented with a “degree of confidence.” Best practice design would entail procedural precautions that would failsafe the system and make a prediction impossible when the system’s confidence is low on a prediction [38]. Unlike human performance, which is prone to significant inter- and intra-individual inconsistency, neural network will generate predictions with little variance and high repeatability, but this should not erroneously be interpreted as high confidence in a result.

### Management of medical data used in AI software for facial imaging

With the use of deep learning in medical diagnostics, dynamic autonomous AI solutions and big data are being introduced to medicine, and conventional ethical and legal principles may therefore not be capable to ensure that all facets of accountability are regulated. In order to uphold the quality of the software and warrant its safeness as a medical tool, it will be necessary to establish new standards and stipulate them in quality management and patient safety plans. These plans must cover a wide variety of issues, and most of them are very acute when working with images of the face. As an example, the feeding of patients’ facial image in a neural network will raise a multitude of ethical and juridical concerns [39], among them:

1. *informed consent:* should medical professionals disclose that the diagnostic outcome of multi-layer convolutional networks are not fully explainable?
2. *data privacy*: should the medical professional disclose that the image of the patient and the obtained prediction will be used to further train the network?
3. *data security and cybersecurity:* facial images are legally considered “particularly sensitive personal data” [40]. Thus, how can the required higher standards of privacy protection be met?
4. *liability:* would a detrimental treatment based on an incorrect prediction of an AI-software be charged for medical malpractice (i.e., the dentist) or product liability (i.e., the company) [3]?
5. *intellectual proprietary right*: who owns the data of a continuously evolving AI software? And may the owner dispose of the data freely?

While some have voiced that the demands for health care privacy may no longer be attainable with the enormous scale of data sharing [3], there is no question that the wider public is uncomfortable with the prospect of companies or even the government selling patient data for profit, sometimes for billions [41]. These are not hypothetical considerations. For example, the Royal Free NHS Foundation trust was found to have breached laws of data privacy when it shared personal data of 1.6 mio. patients to Google Deep Mind for the development of an app to diagnose acute kidney injury [39].

## Necessary improvements

### Interpretability and explainable AI

**Fig. 4:**
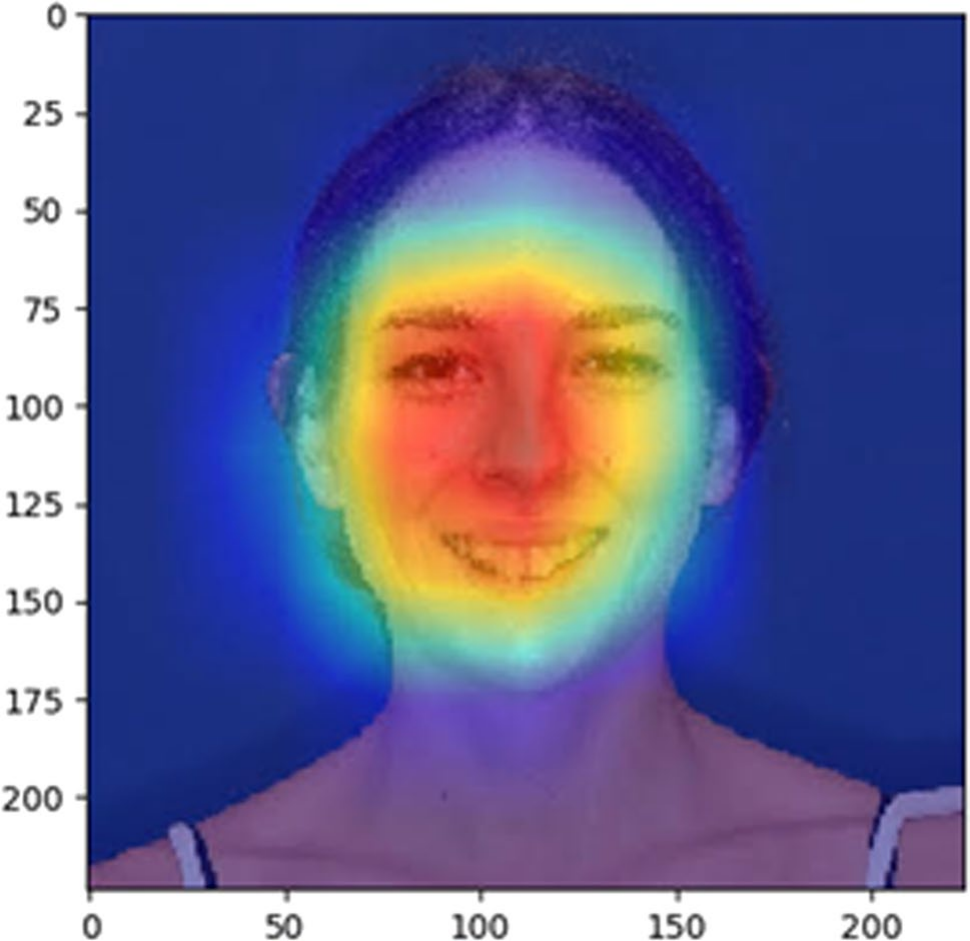
Data visualization method (“heat map”) based on Grad-CAM (https://arxiv.org/abs/1610.02391) to identify discriminative regions by magnitude, used by the convolutional neural network (CNN) for attractiveness of the face. As can be seen, it is unrealistic to assume that the heat map would be able to point to such fine-grained details and disclose on which the network focuses

In machine learning, there is usually a trade-off between accuracy and interpretability: Rule-based systems are highly interpretable but not very precise. Using deep models, interpretable rules are traded for complex algorithms that achieve superior performance through greater abstraction (more layers) and tighter integration (end-to-end training). Thus, recently introduced deep networks are built sometimes on an uninterpretable amount of 200 layers in order to achieve a state-of-the-art performance in a variety of challenging tasks [42]. As such, the system is built to produce an inscrutable prediction that is not open to inspection or interpretation. To mitigate this so-called black box decision-making, explainable AI (XAI), or interpretable AI, aims to establish more transparency by making the results of the solution comprehensible to humans. For predictions based on image analysis, a broadly used approach in XAI is class activation mapping, i.e., generating a sort of heat map. The idea is to identify exactly which regions of an image are being used for discrimination [43]. Such heat maps have been advocated for medico-dental imaging [28] and have indeed already been applied to the analysis of cephalometric [44] or panoramic radiography [45]. The use of heat maps in image analyses of the face, be it for the diagnostic evaluation of attractiveness, gender, or age, would—at least in theory—allow to increase the interpretability of the system and confidence in the prediction [17]. But even with newest techniques of visual explanation, it is unrealistic to assume that the heat map is able to point to such fine-grained details that built the prediction for attractiveness, age, or gender (Fig. 4) [42].

### Regulatory oversight

Expanding on the concerns outlined above related to the translation of commercial software to medical application, it seems crucial to establish regulation processes to overcome the obstacles associated to lack of transparency or to bias inherent in the data. Moreover, the algorithms should be monitored for their performance, robustness, and resilience to withstand changing clinical inputs and conditions [46].

Both the European Medicine Agency (EMA) and the US Food and Drugs Administration (FDA) have addressed the necessity to establish legal and ethical regulation processes in licensing AI-based medical devices. In a bold step, the FDA has acknowledged that the current regulations are not up to par for the modern techniques, and while the administration pro-actively presents in a published action plan concrete steps it intends to undertake [47], it also reaches out and requests feedback from the community [48]. In its action plan, the FDA proposes among other changes probably most importantly the introduction of a good machine learning practice scheme, the establishment of a public repository for approved AI-based software, and the revision of the regulatory oversight. The EU and the FDA stipulate that regulation and approval of AI-based software as medical devices should be effected according to the risk categorization described in the Medical Device Regulation (MDR), which came into effect in 2021 [39]. This regulation considers even a software that is solely used for the medical purpose of *prediction or prognosis of disease* as a medical device. The recommended categorization by MDR differentiates between the state of health care situation (critical, serious, non-serious), the intended use of the software-based information, and the level of impact (treat or diagnose, drive clinical management, mitigating public health). The use of most if not all AI-based algorithms for the face presented in this review would probably be considered to diagnose or drive clinical management of serious but non-critical cases.

Yet, the most serious impediment in regulatory oversight is the very essence of what makes AI so exceptional. The forte of neural networks is their ability to constantly improve by means of real-world training. As much as this perpetual learning process based on highly iterative and adaptive algorithms has made these technologies uniquely situated among software and has helped to open countless new avenues, it is exactly this autonomy that has created a novel state of affair that current regulatory frameworks were not designed for and are struggling to handle. In the past, the FDA has therefore mostly approved algorithms that were “locked” prior to marketing (meaning the algorithm is tampered and provides the identical outcome each time the same input is applied to it and does not change when exposed to real-world feedback) [48]. This wing-clipping is however absurd, as it literally removes the “intelligence” of AI, and it seriously curtails the inherent autonomy and the desired capability of AI-based software to learn and adapt in a real-world environment. Currently, the FDA is working on a framework described as “Predetermined Change Control Plan.” This plan intends to pre-define already in the conception phase the various anticipated modifications together with the associated methodology to be used, to allow these changes in a controlled manner [47]. Such a plan would enable to monitor an adaptive software product from its premarket development through post-market performance. This strategy would indeed provide a certain degree of assurance of safety and effectiveness while still acquiescing to the autonomy of artificial intelligence in medical devices. Time will tell whether the FDA will be able to have its cake and eat it.

## The moral imperative

Any rise of novel technological possibilities in medicine is accompanied by new challenges regarding moral responsibilities that go far beyond the discussed technical and judicial requirements. If a moral compass is lacking, misuse cannot be prevented, as any regulations will be viewed as just an obstacle course. So, while thorough regulations are indisputably of great importance, and while the application of AI in medicine can be promoted to advance science or even the profit of shareholders, it should be guided and dictated by moral accountability. Everyone involved in the development of AI solutions in medicine is called upon this moral imperative.

As a case in point, current law largely fails to address discrimination when extracting information from big data [35, 39]. Trained to perform certain diagnostic or interventional tasks according to a predetermined goal, the AI solution will depend immensely on the input. Yet, the input is not value free. Let us consider the use of attractiveness—instead of beauty—to score medical images. Attractiveness is defined as the quality of being pleasing, and unlike beauty, attractiveness does not describe the face per se, but rather how the face is being perceived. This facilitates the task, because attractiveness can easily be rated and ranked by observers (i.e., perceiver), and these many observations can be fed to CNN. But it is vital to appreciate the fact that the ratings are not value free, as a specific *combination of people being scored and people scoring the images is actually setting a norm*. The use of a multitude of subjective opinions with big data will not make the score any more objective or free of bias. The bias of the individual will be replaced by the bias of the group, and a whole group might be biased toward a certain ethnicity, skin color, or lip volume.

Therefore, applying AI toward personalized medicine could also be considered a step in the wrong direction. Personalized orthognathic surgery, for example, would in many instances make faces less deviating, but not ideal. Striving for the highest attractiveness score might be incompatible with an individual’s perceived idea of beauty and might cause negative psychological effects owing to estrangement.

Research groups, software developers, shareholders, and practitioners all have a moral obligation to recognize that feeding big data to AI solution is a balancing act, and that prior to any endorsement, all involved people are called upon to question the moral quality of new norms that will inevitably be set with advancement of AI applications in medico-dental diagnostics.
